# Golgin45-Syntaxin5 Interaction Contributes to Structural Integrity of the Golgi Stack

**DOI:** 10.1038/s41598-019-48875-x

**Published:** 2019-08-28

**Authors:** Neeraj Tiwari, Morven Graham, Xinran Liu, Xihua Yue, Lianhui Zhu, Dipak Meshram, Sunkyu Choi, Yi Qian, James E. Rothman, Intaek Lee

**Affiliations:** 1 Department of Cell Biology, Yale University School of Medicine, New Haven, CT 06520 USA; 2 School of Life Science and Technology, ShanghaiTech University, Shanghai, China

**Keywords:** Golgi, Golgi

## Abstract

The unique stacked morphology of the Golgi apparatus had been a topic of intense investigation among the cell biologists over the years. We had previously shown that the two Golgin tethers (GM130 and Golgin45) could, to a large degree, functionally substitute for GRASP-type Golgi stacking proteins to sustain normal Golgi morphology and function in GRASP65/55-double depleted HeLa cells. However, compared to well-studied GM130, the exact role of Golgin45 in Golgi structure remains poorly understood. In this study, we aimed to further characterize the functional role of Golgin45 in Golgi structure and identified Golgin45 as a novel Syntaxin5-binding protein. Based primarily on a sequence homology between Golgin45 and GM130, we found that a leucine zipper-like motif in the central coiled-coil region of Golgin45 appears to serve as a Syntaxin5 binding domain. Mutagenesis study of this conserved domain in Golgin45 showed that a point mutation (D171A) can abrogate the interaction between Golgin45 and Syntaxin5 in pull-down assays using recombinant proteins, whereas this mutant Golgin45 binding to Rab2-GTP was unaffected *in vitro*. Strikingly, exogenous expression of this Syntaxin5 binding deficient mutant (D171A) of Golgin45 in HeLa cells resulted in frequent intercisternal fusion among neighboring Golgi cisterna, as readily observed by EM and EM tomography. Further, double depletion of the two Syntaxin5-binding Golgin tethers also led to significant intercisternal fusion, while double depletion of GRASP65/55 didn’t lead to this phenotype. These results suggest that certain tether-SNARE interaction within Golgi stack may play a role in inhibiting intercisternal fusion among neighboring cisternae, thereby contributing to structural integrity of the Golgi stack.

## Introduction

The Golgi apparatus is a central sorting station that orchestrates membrane transport at the cross-road of the secretory and the endocytic pathways^1^. The biogenesis of the Golgi apparatus is known to be mediated by a group of Golgin tethers and two Golgi Reassembly and Stacking proteins (GRASPs; GRASP65/55), and the Golgi undergoes dynamic disassembly and reassembly during mitotic cell division^2–6^. GRASPs have been shown to mediate Golgi stacking by their PDZ domain interaction *in trans*^7,8^. On the other hand, Golgins, such as GM130, seem to be essential for SNARE-mediated membrane fusion of mitotic Golgi membranes for regeneration of Golgi cisternae during post-mitotic Golgi reassembly^9,10^. However, it is unclear by what mechanism GM130 directly contributes to cisternal stacking.

We recently reported that both GM130 and Golgin45 can substitute for GRASPs to such an extent that their exogenous over-expression can create functionally normal Golgi stacks in GRASP65/55-depleted mammalian cells, suggesting that the two Golgins and GRASPs play extensively complementary roles under physiological conditions^11^. This is consistent with the findings that GRASP homologs, *Grh1* and dGRASP, are each largely dispensable for Golgi stacking in yeast *S*.pombe and drosophila S2 cells, respectively^12,13^.

GM130 utilizes its N-terminal domain (proximal to its Cdc2 phosphorylation site) to bind to another Golgin tether, p115^14,15^ and to Golgi *t*SNARE Syntaxin5 via its coiled-coil domain 4–6 (CC4-6)^15^. GM130 also interact directly with Rab1-GTP (using CC1-3) and Rab33b-GTP (CC4-6)^16–18^. These interactions are known to play important roles in coordinating SNARE-mediated membrane fusion of incoming ER-derived cargo carriers to *cis*-Golgi cisternae^17^, but precise binding interactions of Syntaxin5 and these Rab-GTPases with GM130 are poorly understood^15^. Golgin45 is known to bind Rab2-GTP and ACBD3 via its central coiled-coil region and GRASP55 via its C-terminal PDZ-binding motif^3,7,19^, but the exact mechanism by which Golgin45 contributes to Golgi structure has remained elusive.

In this study, we report a novel interaction between Golgin45 and a Golgi tSNARE, Syntaxin5. We further demonstrate using EM and EM tomography that this protein-protein interaction between the two Golgi matrix components significantly contributes to structural integrity of the Golgi stack by inhibiting intercisternal fusion between neighboring Golgi cisterna.

## Results and Discussion

### Golgin45 is a syntaxin5-binding Golgin tether

**Figure 1:**
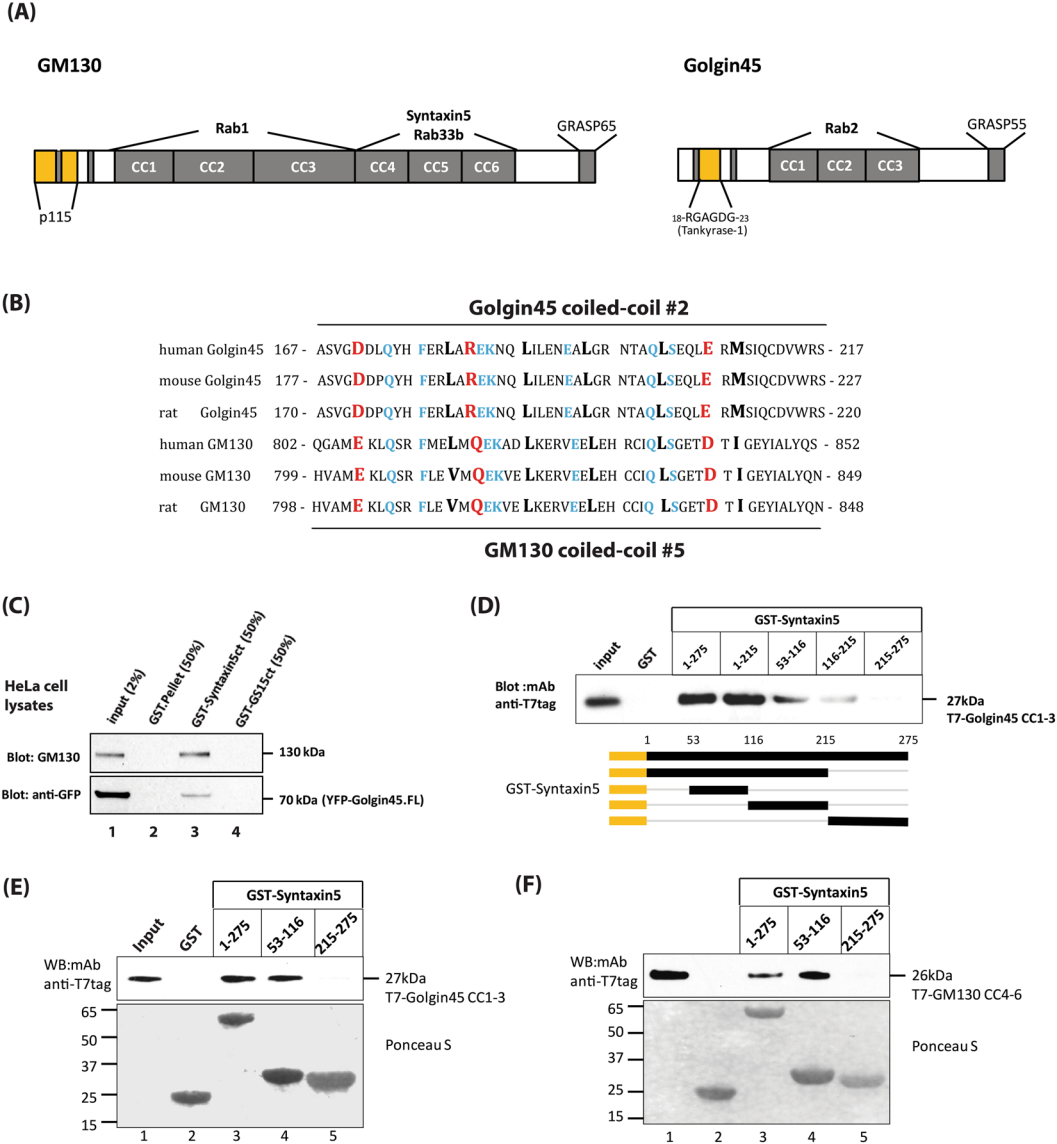
Golgin45 and GM130 share a common leucine zipper-like motif that is likely to play a crucial role in their binding to Golgi tSNARE, Syntaxin5. (**A**) Illustration indicating the domain arrangement and positions of important protein binding sites found in GM130 and Golgin45, respectively. (**B**) Sequence alignment between GM130 and Golgin45 CC domains, containing Leucine zipper-like motifs; red indicates conserved charged residues; light blue indicates strictly conserved residues; bold black indicates leucine zipper-like motifs. (**C**) Cytoplasmic domain of Syntaxin5 captures GM130 and YFP-Golgin45 from HeLa cell extract in GST pull-down assays. GST-GS15 was used here as a negative control. (**D**) Domain mapping of Golgin45 binding site on Syntaxin5 by GST pull-down assays show that the N-terminal regulatory domain, but not the SNARE motif, is responsible for their direct interaction. (**E**,**F**) GST pull-down assays showing direct binding between purified recombinant CC domains (Golgin45 CC1-3 and GM130 CC4-6, respectively) and GST-Syntaxin5 full length cytoplasmic domain (AA1-275) or the H3 domain (AA53-116), but not the SNARE motif (AA215-275). Both Golgin45 and GM130 CC domains showed significant binding to the H3 domain.

In order to characterize the functional role of Golgin45 on Golgi structure, we first postulated that Golgin45 could be a functional homologue of GM130, based on its interaction with GRASP55 (GRASP65 for GM130) and Rab2-GTP (Rab33b-GTP for GM130). This assumption led us to hypothesize that, like GM130, Golgin45 may also bind Golgi tSNARE, Syntaxin5, using its Rab-binding domain. Upon close examination of amino acid sequence homology, we found highly conserved leucine zipper-like motifs, commonly shared by GM130 (CC5) and Golgin45 (CC2) (Fig. 1A,B). As this region of GM130 (CC4-6) had previously been implicated in binding Syntaxin5 directly^15^, we posited that CC2 of Golgin45 could interact with Syntaxin5 as well. Initially, we used GST pull-down assays to test whether Syntaxin5 can bind both GM130 and Golgin45 from HeLa cell extract. The results showed (Fig. 1C) that GST-Syntaxin5, but not GST-GS15, captured GM130 and YFP-Golgin45 from HeLa cell extract, suggesting that both Golgins might bind Syntaxin5 directly.

### N-terminal regulatory domain (H3) of Syntaxin5 interacts with Golgin45

As individual CC domains of both GM130 and Golgin45 were highly unstable in solution upon purification, 6xHis-tagged recombinant GM130 (CC4-6) and Golgin45 (CC1-3) were expressed and purified from BL21 to further study their binding interaction to Syntaxin5 and its truncation mutants. The results showed that the N-terminal regulatory domain (H3) of Syntaxin5 (amino acids (AA) 1-215) is likely to be responsible for its binding to Golgin45 (CC1-3), whereas the SNARE domain (AA215-275) failed to show any binding (Fig. 1D,E). Binding of recombinant GM130 (CC4-6) to GST-Syntaxin5 showed a similar pattern (Fig. 1F), suggesting that GM130 and Golgin45 seem to use the leucine zipper-like domain for binding to Syntaxin5 H3 domain.

### The D171A mutation in Golgin45 CC2 abrogates its binding to syntaxin5

**Figure 2:**
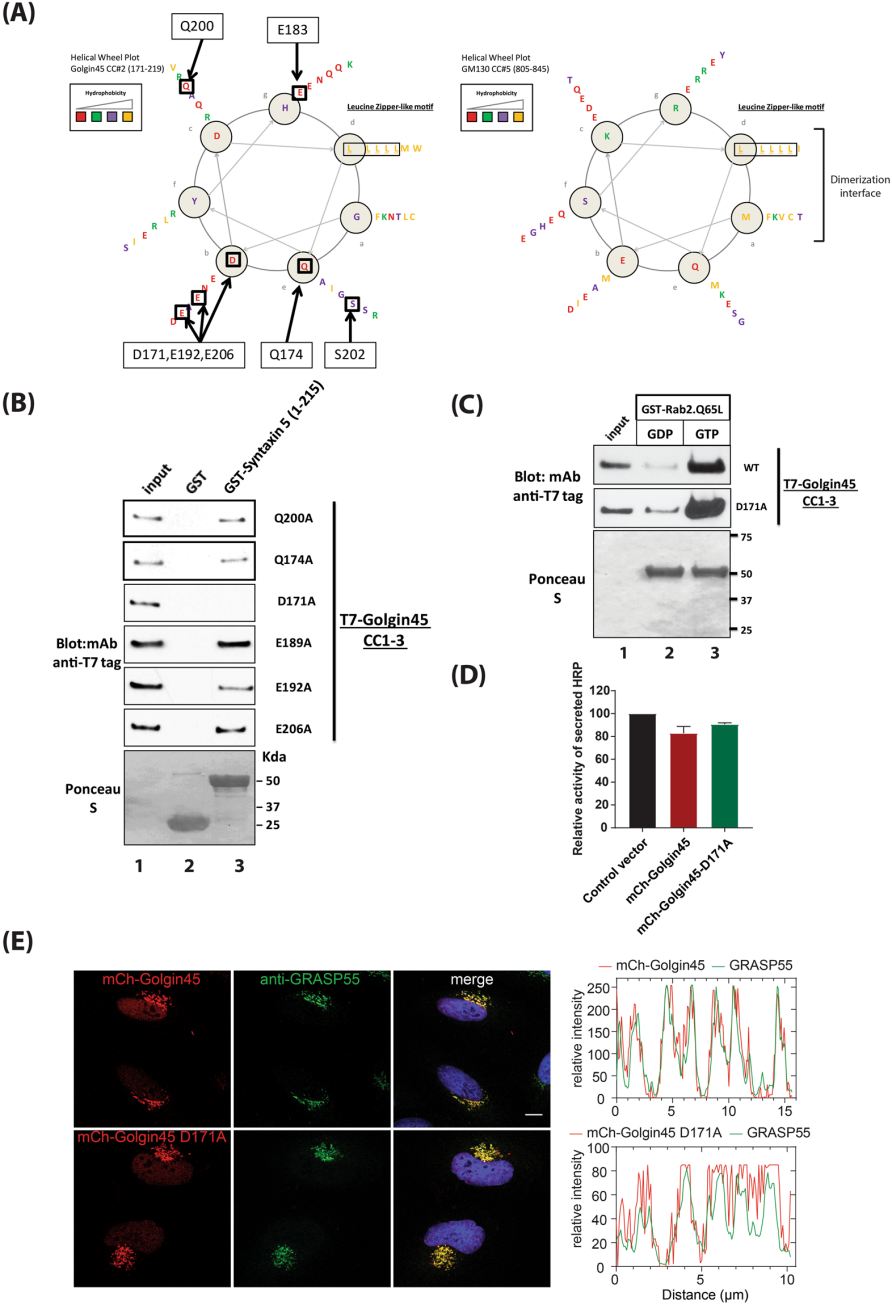
Alanine scanning mutagenesis of Golgin45 Leucine zipper motif reveals that D171A mutant abrogates Golgin45 binding to the N-terminal regulatory domain of Syntaxin5, but not to Rab2-GTP. (**A**) Helical wheel plots showing the positions of homologous amino acid residues found in the leucine zipper-like motifs of GM130 and Golgin45. Boxed residues indicate identical or similar AA residues; (**B**) Alanine scanning mutagenesis of common AA residues within Leucine zipper motif of Golgin45 and GST pull-down assays were used to identify a specific point mutant of Golgin45 that fails to bind recombinant Syntaxin5. D171A mutation significantly abrogates Golgin45 CC domain binding to GST-Syntaxin5 (1–215) in pull-down assays. (**C**) GST-Rab2.Q65L recombinant proteins were used to test if Golgin45.D171A mutant show any change in their interaction, compared to the WT Golgin45. Both the WT and D171A.Golgin45 equally bound to Rab2 in GTP-dependent manner, suggesting Syntaxin5-binding deficient mutant is not affected in Rab2 interaction *in vitro*. (**D**) Expression of mCherry-Golgin45.D171A does not significantly affect secretion of soluble secretory cargo, ss-HRP, in HeLa cells, compared to the cells transfected with vector-transfected control, while expression of mCherry-Golgin45 WT very moderately inhibits secretion of ss-HRP. (**E**) D171A mutation does not affect Golgin45 targeting to the Golgi and its co-localization with endogenous GRASP55 in HeLa cells, compared to that of Golgin45 WT. Line analysis graph shows that both the WT and the mutant mCherry-Golgin45.D171A co-localize well with anti-GRASP55 stained Golgi area. Bar = 10 μm.

To further characterize the binding interaction, alanine scanning mutagenesis of Golgin45 CC2 was carried out to identify a point mutation that abrogates Golgin45-Syntaxin5 interaction (Fig. 2A; see boxed amino acid residues in the helical wheel plots). We then performed GST pull-down assays using the purified recombinant mutant proteins and GST-Syntaxin5 N-terminal domain (1–215). The results showed that D171A mutation causes a significant reduction in Golgin45-Syntaxin5 binding, but not in the interaction between Rab2-GTP and Golgin45 (Fig. 2B,C).

To examine the effect of this mutation on Golgi function, we co-transfected HeLa cells with a soluble secretory cargo (ss-HRP) and either mCherry tagged Golgin45 WT or Golgin45.D171A mutant for 18 hours. We then used ELISA assays to detect secreted ss-HRP in the conditioned media. The results (Fig. 2D) showed that expression of either mCherry-Golgin45 WT or the mutant Golgin45 resulted in moderate reduction in the amount of ss-HRP in the conditioned media, compared to vector-transfected control cells, suggesting that anterograde protein secretions are not significantly affected in these cells.

Confocal results suggested that both mCherry-Golgin45 WT and the mutant mCherry-Golgin45.D171A were correctly targeted to the Golgi and co-localized well with endogenous GRASP55 (Fig. 2E), demonstrating that Syntaxin5 binding may not be important for Golgin45 targeting to the Golgi.

### Expression of the Golgin45 D171A mutant results in inter-cisternal fusion

**Figure 3:**
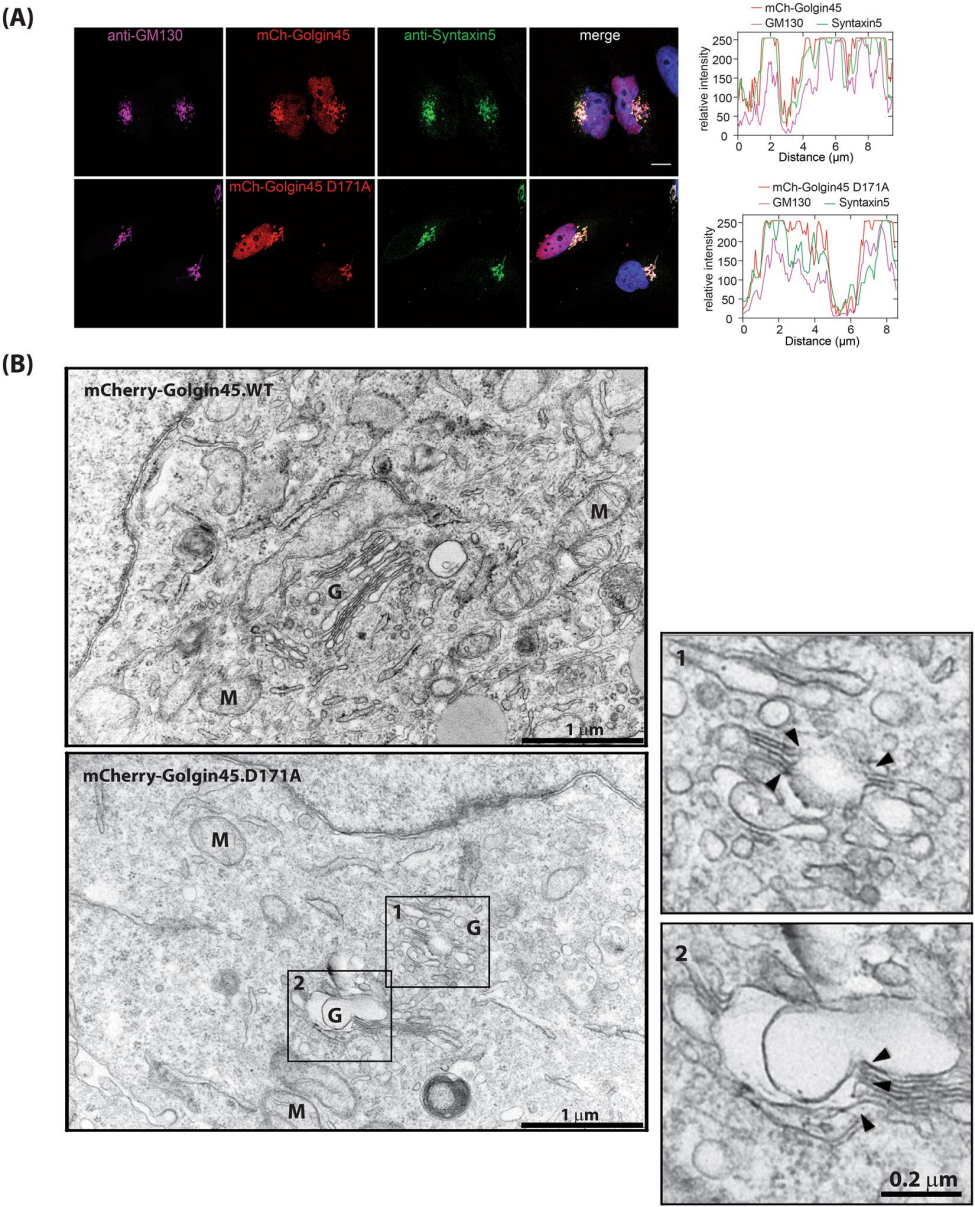
Exogenous expression of Golgin45.D171A mutant results in intercisternal fusion. (**A**) Both WT Golgin45 and Golgin45.D171A mutant significantly co-localized with endogenous Syntaxin5 in HeLa cells. GT-GFP was co-expressed as a Golgi marker. Bar = 10 μm (**B**) Representative EM photo showing that expression of Golgin45.D171A mutant, but not the Golgin45 WT, results in frequent intercisternal fusions at the Golgi in HeLa cells. Insets show a magnified view of the boxed area. Intercisternal fusions are marked by black arrow heads. (scale bar = 1 μm) M = mitochondria, N = nucleus, G = Golgi.

In order to test whether expression of the Golgin45 D171A mutant might influence Syntaxin5 localization to the Golgi, we transfected mCherry-tagged Golgin45 WT or D171A mutant in HeLa cells for 18 hours. The cells were then fixed and stained with anti-GM130 (Golgi marker) and anti-Syntaxin5 antibodies for examination under confocal microscope. The results (Fig. 3A) showed that expression of Golgin45.D171A mutant had no obvious effect on Golgi structure and Syntaxin5 localization to the Golgi at light microscope level.

These cells were then processed for EM to further study any alteration of the Golgi structure at EM resolution. Strikingly, we found that exogenous expression of the D171A mutant, but not the WT Golgin45, resulted in frequent intercisternal fusions among Golgi cisterna (Fig. 3B), suggesting that this tether-SNARE interaction may be important for structural integrity of the Golgi stack, although we cannot rule out the possibility that this particular mutation (D171A) may disrupt some other important protein-protein interaction(s), leading to the observed phenotype.

### The Golgin45 D171A mutant fails to restore normal Golgi morphology in GRASP-double depleted heLa cells

We had previously demonstrated that exogenous expression of WT Golgin45 restores normal Golgi stack morphology and secretory function in GRASP65/55 double-depleted cells^11^. We used this experiment as an assay to test the hypothesis that, if Golgin45-Syntaxin5 interaction is crucial for Golgi structural integrity, the mutant Golgin45 (D171A) may fail to restore normal Golgi morphology in GRASPs double-depleted cells. We treated HeLa cells with siRNAs against human GRASP65 and GRASP55 for 48 hours, followed by transfection with either mCherry-Golgin45 WT (control) or mCherry-Golgin45 D171A mutant for 18 hours^11^. The cells were then processed for electron microscopy.

**Figure 4:**
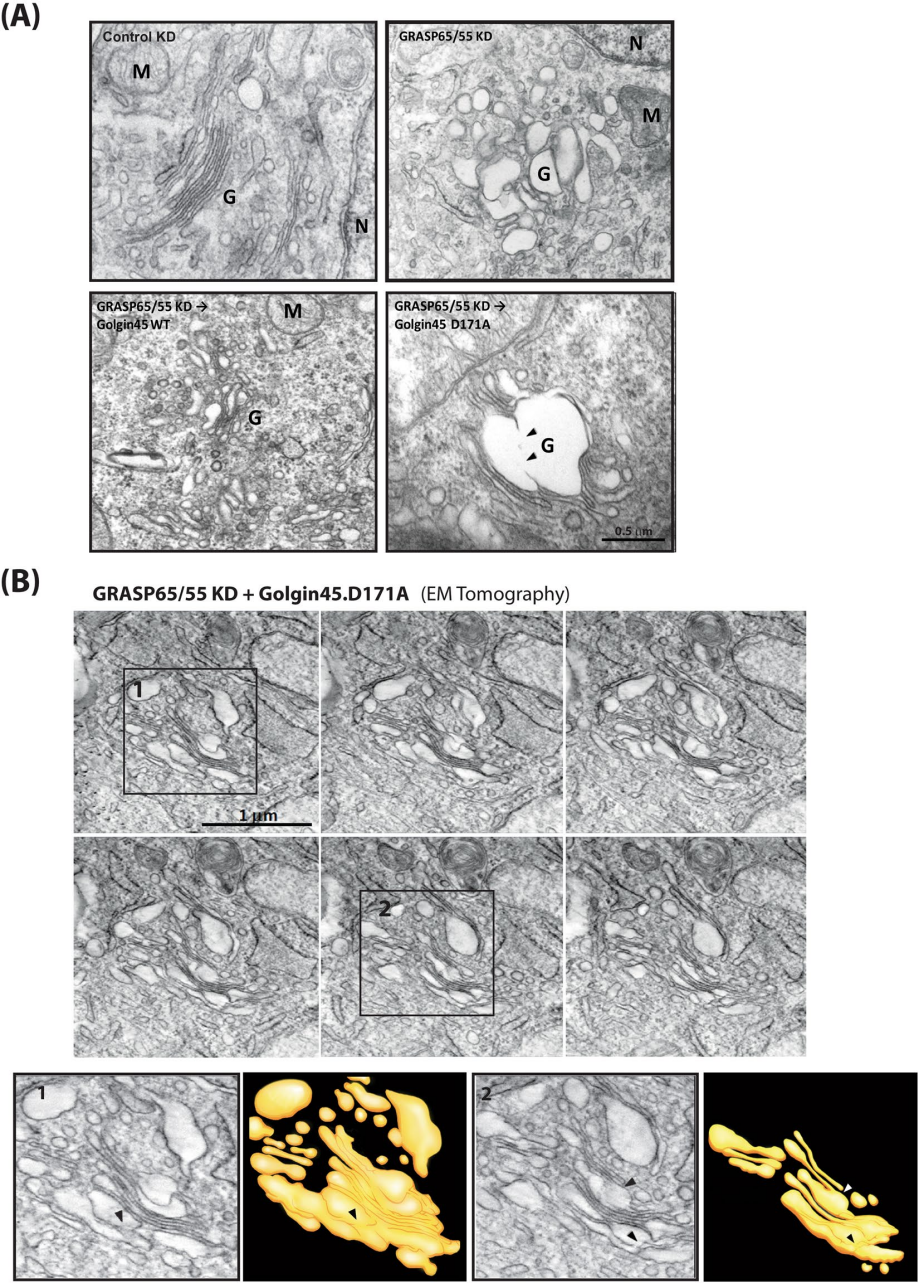
Electron tomography of the Golgi showing extensive intercisternal fusion induced by Golgin45.D171A expression. (**A**) Golgin45.D171A mutant fails to restore normal Golgi morphology in GRASP65/55-depleted cells, whereas expression of WT Golgin45 successfully restores normal Golgi stack morphology, as shown previously. (**B**) EM tomography tilt series showing extensive intercisternal fusion occurring throughout the Golgi stack in HeLa cells treated with GRASP65/55 siRNAs for 48 hours, followed by overnight expression of Golgin45.D171A mutant. Insets show the boxed areas along with 3D reconstruction of inter-cisternal fusion sites. Black arrow heads indicate intercisternal fusions.

The results showed that, as expected, exogenous expression of WT Golgin45 restored normal Golgi stack morphology (Fig. 4A; bottom left panel) to a significant extent in these cells, although the cisterna in the restored Golgi were still moderately dilated and the Golgi ribbon remained fragmented. However, expression of the D171A mutant not only failed to restore Golgi morphology, but also resulted in frequent intercisternal fusion among the severely dilated Golgi cisterna (Fig. 4A; bottom right panel). Due to significant membrane fusion among the neighboring cisternae, it was difficult to quantify this change by measuring maximum cisternal width, as was done in our previous study^11^.

### EM tomography further confirms inter-cisternal fusion, induced by expression of the Golgin45 D171A mutant

To further support these findings, we performed EM tomography to obtain higher quality images of fused Golgi cisterna at EM resolution. HeLa cells were treated with GRASP65/55 siRNAs, followed by expression of the mCherry-Golgin45 D171A mutant for 18 hours. The EM tomograph images from these experiments indeed confirmed that there are extensive intercisternal fusions throughout the Golgi stacks in these cells (Fig. 4B; movie #1). Taken together, these results indicate that Golgin45-Syntaxin5 interaction may have a role in structural integrity of the Golgi stack.

### Individual knock-down of Golgin45 or GRASP55 leads to a reduction in the average number of cisternae per Golgi stack but does not result in inter-cisternal fusion

**Figure 5:**
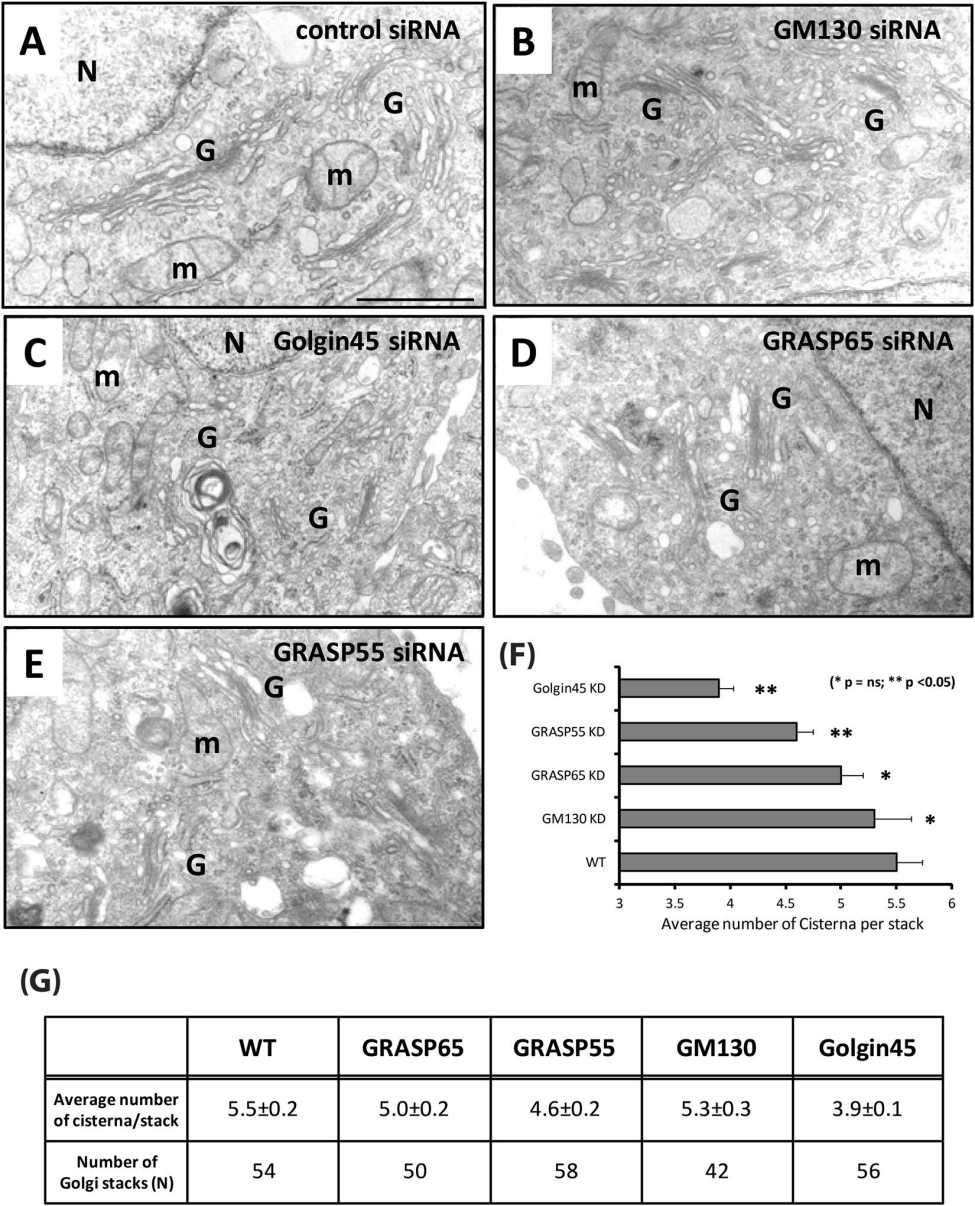
Depletion of Golgin45 leads to a significant reduction in the number of cisternae per Golgi stack (**A**–**E**) Representative EM photos showing HeLa Golgi stacks in Golgin or GRASP single knowndown cells. HeLa cells were treated with siRNAs against scrambled control (**A**); GM130 (**B**); Golgin45 (**C**); GRASP65 (**D**); GRASP55 (**E**) for 96 hours and processed for EM study, as described previously. (**F**) Bar graph summarizing the average number of cisternae per stack in single KD cells with standard deviation; (**G**) Table summarizing the results and the number of Golgi stacks used in the quantification for the number of cisternae per stack. (scale bar = 1 μm) M = mitochondria, N = nucleus, G = Golgi.

Interestingly, we failed to observe intercisternal fusion in HeLa cells treated with Golgin45 siRNAs for 96 hours. Nor did we find intercisternal fusion in GM130 or GRASP-depleted cells (Fig. 5A–E). However, we did find significant reduction in the average number of cisternae per stack in Golgin45 KD cells and GRASP55 KD cells (Fig. 5F,G; also see histograms in Supplementary Fig. 1), suggesting that these two medial Golgi stacking proteins may play the most critical role for Golgi structure.

### Simultaneous depletion of the two Syntaxin5-binding Golgins results in inter-cisternal fusion

**Figure 6:**
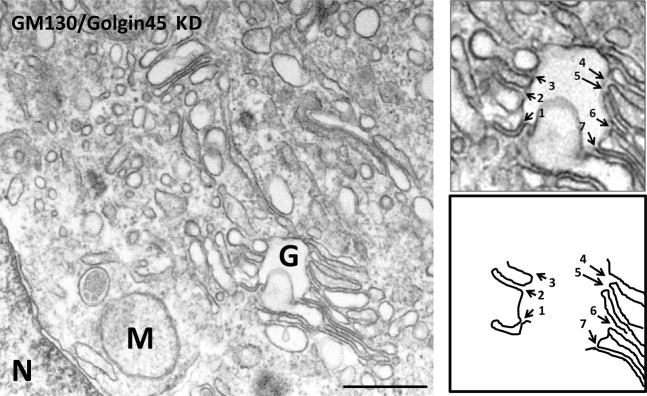
Double knockdown of GM130/Golgin45, but not GRASP65/55 results in intercisternal membrane fusion between neighboring Golgi cisternae. Representative EM micrograph of HeLa cells treated with siRNAs against GM130/Golgin45 siRNA for 96 hrs (scale bar = 0.5 μm). Double knockdown of the two Syntaxin5-binding Golgins resulted in significant intercisternal fusion among the Golgi cisterna, as indicated by tracing lines for Golgi membranes in the inset. In this photo, at least 3–4 Golgi cisterna have fused with one another to form a Golgi stack with extensively continuous luminal space. M = mitochondria, N = nucleus, G = Golgi apparatus.

As these Golgins and GRASP-type proteins seem to be capable of functionally substituting for one another, we then decided to examine the effect of Golgin double KD or GRASP65/55 double KD to see whether double depletion of either the two Syntaxin5-binding Golgins or the GRASPs may lead to inter-cisternal fusion. Interestingly, simultaneous depletion of GM130/Golgin45 (Fig. 6) resulted in massive fusion among 3–4 neighboring cisterna, whereas neither the control siRNA-treated nor GRASP65/55 depleted cells (see Fig. 4A; upper panel) resulted in this phenotype.

We routinely found various degrees of inter-cisternal fusion in ~20% of GM130/Golgin45 double-depleted HeLa cells examined under EM. Again, it was difficult to systematically quantify inter-cisternal fusion, partly due to highly diverse morphologies of the fused Golgi membranes on these EM photos, which were obtained using thin section EM method that was employed for these experiments.

Syntaxin5 is known to be localized throughout the stacks^20^, as are one or another of Golgin45 and GM130^3,5^. Therefore, this binding interaction may help prevent inter-cisternal fusion through a significant portion of the Golgi stack. In summary, we propose that Golgin45-Syntaxin5 interaction represents a novel protein-protein interaction among the Golgi matrix components, which may contribute to structural integrity of the Golgi stack.

## Methods

### Reagents and antibodies

All common reagents were purchased from Sigma-Aldrich (St.Louis, MO), unless otherwise mentioned. The following antibodies were used: mouse monoclonal GM130 (BD Transduction Laboratories), goat polyclonal GRASP65 (Santa Cruz Biotechnology, CA), rabbit polyclonal GRASP55 (Proteintech inc., Chicago,IL), HRP-conjugated mouse β-actin antibody (Genscript, Piscataway, NJ). Rabbit polyclonal Syntaxin5 (Synaptic system, Goettingen, Germany). Rabbit polyclonal Golgin45 antibody was made by injecting synthetic Golgin45 peptide (AA40-53) conjugated to KLH (GenScript, Piscataway, NJ). All siRNA oligos were purchased from Integrated DNA Technology (Coralville, IA) and the target sequences were as following: human Golgin45 (GCATCATAGTCTTCAGAGTCCATGG), Human Golgin45 (5′UTR for rescue experiments) (CGGAGAAUAAGAAUCUUAGAGGU), Human GM130 (GGACAATGCTGCTACTCTACAACCA), GRASP55 oligo#1 (CTGCGAGAGACCTCAGTCACACCAA), GRASP55 oligo#2 (CCACCAGGAACTACAGGAATTGAAC), GRASP65 oligo#1 (CCTGAAGGCACTACTGAAAGCCAAT), GRASP65 oligo#2 (CTGGGATGTGGCATTGGCT ATGGGT). Rat GM130 cDNA was obtained from Nobuhiro Nakamura (Kyoto Sangyo University, Kyoto, Japan). Human Golgin45 cDNA was purchased from Addgene (Cambridge,MA).

### Cell culture and treatments

HeLa cells were grown in DMEM supplemented with 10% FBS (Invitrogen, Carlsbad, CA) at 37 C. For transfection with siRNA, cells were plated onto 6-well plates 24 hours prior to transfection. We performed the transfection using Dharmafect-1 (HeLa) (Dharmacon, Boulder, CO), according to manufacturer’s instruction. cDNA transfection was done using standard protocol using Lipofectamine2000 (Invitrogen, Carlsbad, CA) or FugeneHD (Promega, Sunnyvale, CA). Confocal images were obtained using LSM880 confocal microscope (Carl Zeiss, Dublin, CA).

### Immunofluorescence staining

Cells were fixed in 4% PFA in PBS for 15 min at room temperature (RT), followed by permeabilization in 0.3% Triton X-100 in PBS for 3 min. After 3 times washing with PBS, cells were then blocked in blocking buffer containing 2% BSA, 0.05% Triton X-100 in PBS for 30 min at RT. Primary antibodies were diluted in blocking buffer, according to manufacturer’s instruction and incubated with cells for 30 min at RT. Secondary antibodies conjugated to Alexa dyes were diluted in blocking buffer and incubated with cells for 15 min at RT.

### SS-HRP secretion assay

HeLa cells were co-transfected with pcDNA3.1-ss-HRP plasmid plus pC4-mCherry (vector control), pC4-mCherry-Golgin45 WT or D171A mutant plasmids, respectively, using Lipofectamine 3000 (Life Technologies). The conditioned media were harvested 18 hours post-transfection. HRP activity was measured using 1-Step Ultra TMB-ELISA (Thermo), according to the manufacturer’s instructions. Experiments were carried out in triplicate samples and repeated twice.

### GST pull-down assays

Recombinant 6xHis-T7-tagged Golgin45 and GM130 CC domains were expressed in BL21 and purified using manufacturer’s standard protocols. We found that GST-rat Syntaxin5 constructs were highly insoluble in various detergent and salt conditions, and eventually opted to use 2% sarkosyl in TBS and probe sonication to break up the aggregated recombinant proteins in the inclusion bodies and diluted the lysates up to 10-fold in protein refolding buffer containing 2% Triton-X 100 and 30 mM CHAPS plus protease inhibitor cocktail (Roche) in TBS, prior to binding to Glutathione sepharose for purification. Typically, we used 50–100 nM recombinant CC domain proteins and incubated with 50 μg purfied GST-Syntaxin5 on Glutathione beads for 3 hrs at 4 C.

### Image analysis on electron micrographs and data presentation

Unless otherwise stated, we used digital electron micrographs in Tiff format and ImageJ software (NIH) to study the number of cisternae per Golgi stack and cisternal luminal width measurement. To comprehensively assess the functional relationship of the GRASPs and the Golgins, a large number of cisternae and stacks in numerous sections were studied extensively. We performed two independent experiments for each dataset in the figures. Frequency distribution histograms from these experiments are shown in Supplementary Fig. 1.

### Sample preparation and image acquisition for electron microscopy/tomography

The transfected cells were fixed in 2.5% gluteraldehyde in 0.1 M sodium cacodylate buffer pH7.4 with for 1 hour. They were then rinsed in 0.1 M sodium cacodylate buffer, scraped and pelleted in 2% agar. Samples were trimmed and post-fixed in 1% osmium tetroxide for 1 hour, en bloc stained in 2% uranyl acetate in maleate buffer pH5.2 for a further hour, rinsed then dehydrated in an ethanol series and infiltrated with resin (Embed812 Electron Microscopy Science) and baked over night at 60 C. Hardened blocked were cut using a Leica UltraCut UC7, 60 nm sections were collected onto formvar/carbon coated nickel grids and stained using 2% uranyl acetate and lead citrate. These were viewed FEI Tecnai Biotwin TEM at 80 Kv. Images were taken using Morada CCD and iTEM (Olympus) software typically at 26,000 x magnification. For electron tomography, 250 nm sections were collected on formvar/carbon copper grids, labeled on both sides with 10 nm gold particles (UtrectUMC). A tomography tilt series was acquired using FEI Express 3D software on an FEI Tecnai TF20 FEG TEM at 200 kV. Images were reconstructed using IMOD software (University of Colorado, Boulder, CO).

## Supplementary information

Supplementary Information

movie #1
