# Supplementary material for: Golgin45-Syntaxin5 Interaction Contributes to Structural Integrity of the Golgi Stack
Source: Sci Rep. 2019 Aug 28;9:12465. doi: 10.1038/s41598-019-48875-x (PMC6713708; doi:10.1038/s41598-019-48875-x)

Supplementary Information

Golgin45-Syntaxin5 Interaction Contributes to Structural Integrity of the Golgi Stack

Neeraj Tiwari[1], Morven Graham[1], Xinran Liu[1], Xihua Yue[2], Lianhui Zhu[2], Dipak Meshram[2], Sunkyu Choi[2], Yi Qian[2], James E. Rothman[1] and Intaek Lee[2,*]

[1]Department of Cell Biology, Yale University School of Medicine, New Haven, CT 06520 USA

[2]School of Life Science and Technology, ShanghaiTech University, Shanghai, China

Supplementary Figure Legends

Supplementary Fig.1 (A-E) Frequency distribution histograms of the number of cisternae per stack for GRASP or Golgin single knockdown HeLa cells.

Supplementary Fig.2-3 Full length images of Western blot results

Supplementary Fig.1

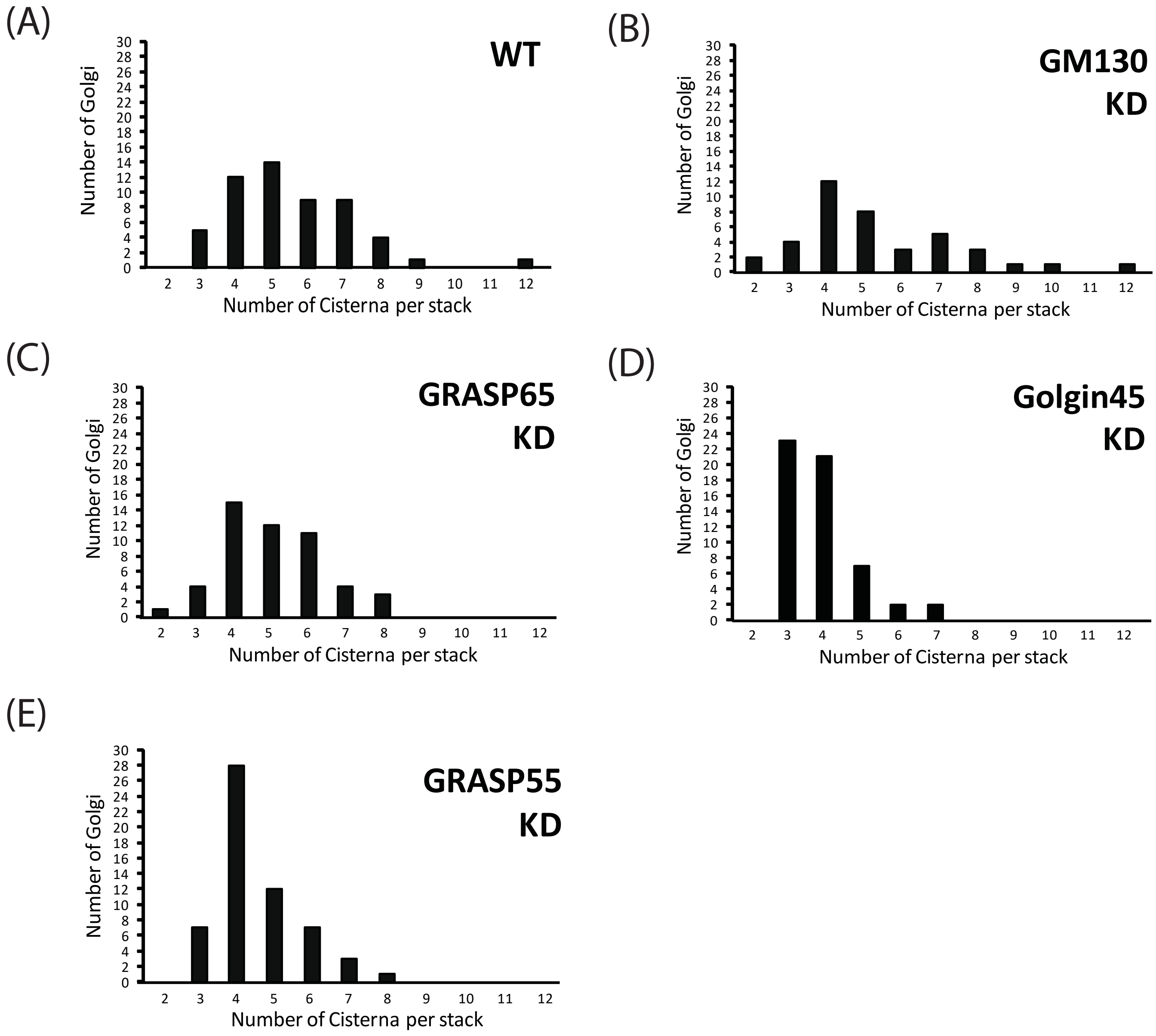

Supplementary Figure 2

Fig.1C

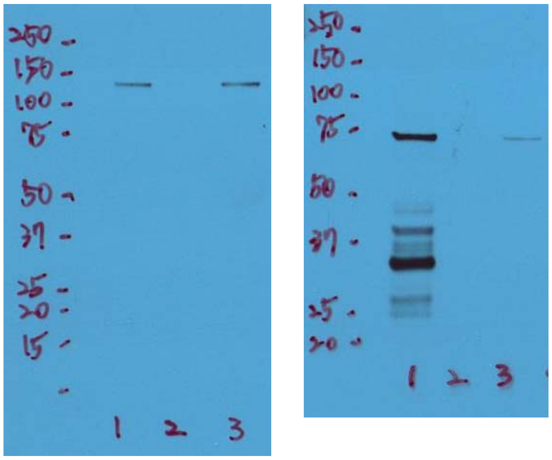

Fig.1D

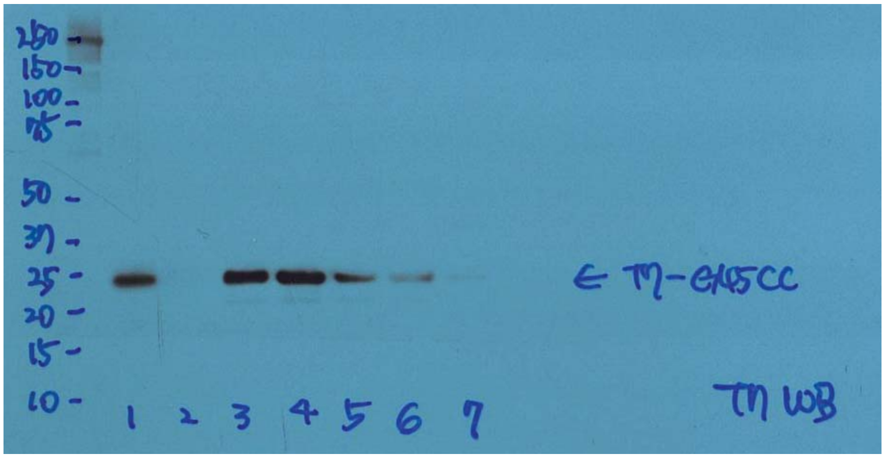

Fig.1E

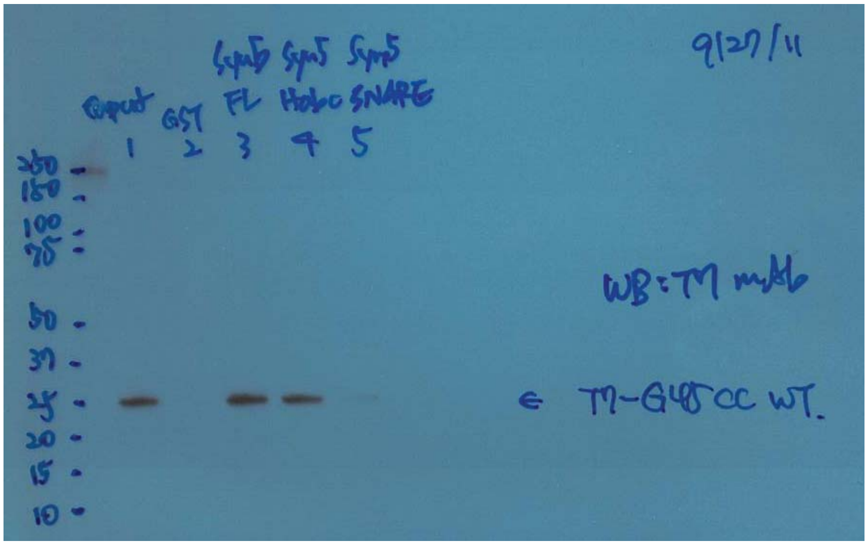

Fig.1F

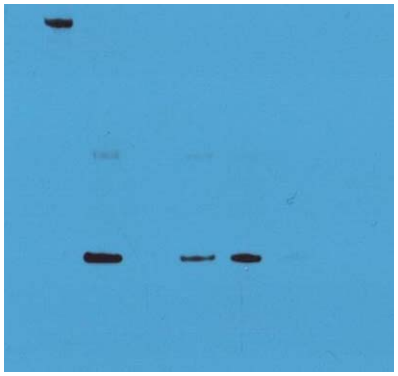

Supplementary Figure 3

Fig.2B

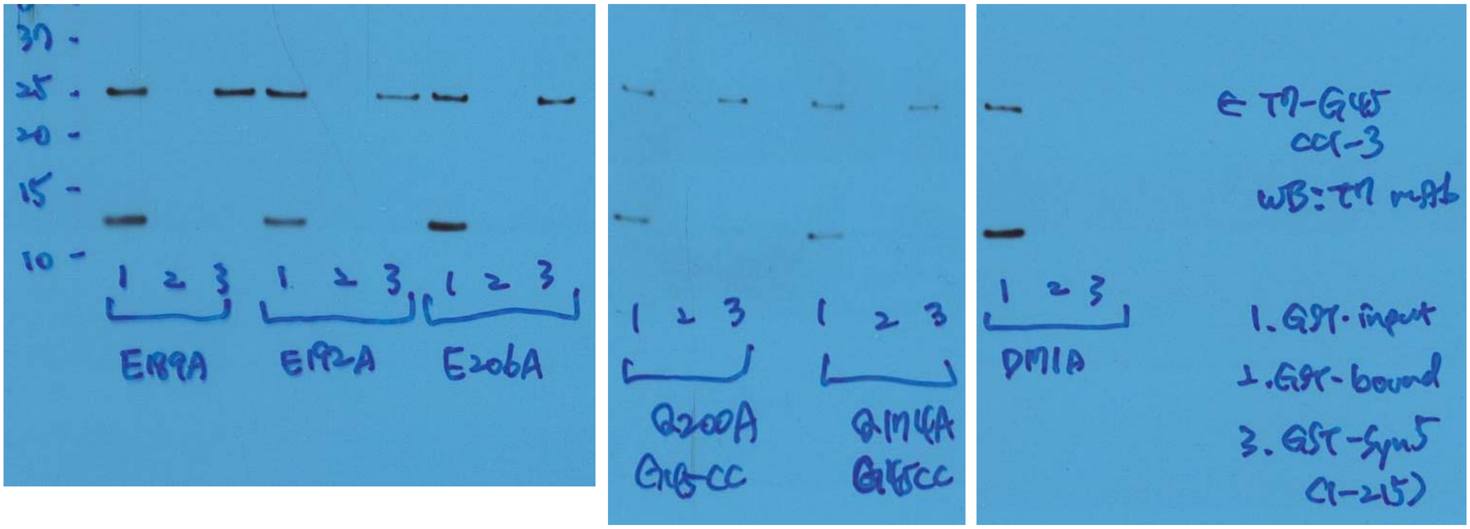

Fig.2C

upper

lower

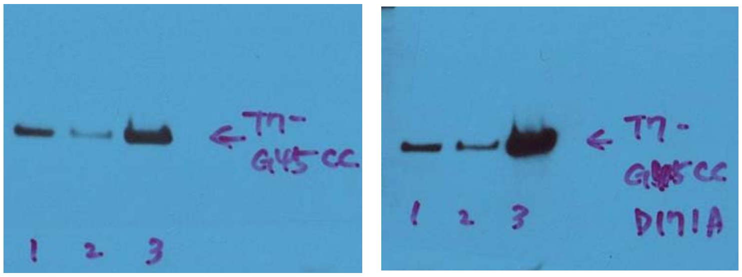

Supplement: Supplementary file 1 — Supplementary Information [file 41598_2019_48875_MOESM1_ESM.pdf]
